# Perceptions of the environmental impact of oncology care: A cross-sectional survey of French and Belgian healthcare professionals

**DOI:** 10.1016/j.joclim.2026.100741

**Published:** 2026-09-10

**Authors:** Paul Matte, Pierre-Florent Petit, Anthony Turpin, Caroline Sarre-Pradon, Adrien Rousseau, Marc Hilmi, Sarah Dumont, Pauline Parent, Anne Rullier, Eva Ester Molina-Beltran, Max Piffoux, Matthieu Delaye

**Affiliations:** a Institut Curie, Department of medical oncology, Paris, France; b CHU HELORA, Department of medical oncology, La Louvière, Belgium; c Lille University hospital, Department of medical oncology, Lille, France; d Gustave Roussy, Department of Medical Biology and Pathology, Villejuif, France; e Paris-Saclay University, Gustave Roussy, Department of Cancer Medicine, Thoracic Group and International Center for Thoracic Cancers (CICT), F-94805, Villejuif, France; f Inserm, Oncostat U1018, labeled Ligue Contre le Cancer, F-94805, Villejuif, France; g Memorial Sloan Kettering Cancer Center, New York, USA; h Gustave Roussy, Department of Medical Oncology, Villejuif, France; i Bordeaux University Hospital, Department of Surgical Pathology, Bordeaux, France; j Institut Curie, Department of medical oncology, Saint-Cloud, France; k Centre Léon Bérard, Department of Medical Oncology, Lyon, France; l Hospices civils de Lyon, Department of Medical Oncology, Lyon, France

**Keywords:** Climate change, Health care sustainability, Environmental impact, Oncology, Cancer care

## Abstract

**Background:**

Health care is a major contributor to greenhouse gas emissions; however, the specific environmental burden of oncology is poorly characterized. This study aimed to assess oncology healthcare professionals’ perceptions of the environmental impact of their clinical practice.

**Methods:**

We conducted an anonymous, cross-sectional survey between January and April 2025 among oncology healthcare professionals in France and Belgium. A 23-item questionnaire was developed by a multidisciplinary team and administered via professional networks, oncology societies, and social media. The survey assessed perceptions of oncology’s environmental impact, attitudes toward sustainable practices, and willingness to modify clinical decisions when supported by scientific evidence.

**Results:**

Among 330 respondents (73% women; median age, 39 years), 32% were medical oncologists, 25% nurses, 8% advanced practice nurses, and 35% other professionals involved in cancer care. Respondents mainly worked in comprehensive cancer centers (45%) or university hospitals (29%). Two-thirds perceived oncology as having a greater negative environmental impact than other medical specialties. Ranked from most to least impactful, the main perceived sources of environmental burden were chemotherapy, biotherapy, radiotherapy, imaging, and patient transport. The integration of environmental considerations into clinical decision-making was low (mean score 8.3/100, SD 14.9), and institutional efforts to reduce impact were perceived as insufficient (mean 15.3/100, SD 20.7). Almost all respondents (97.7%) were willing to implement lower-impact strategies if supported by equivalence data regarding efficacy.

**Conclusion:**

Oncology professionals in France and Belgium recognize that the environmental footprint of cancer care is insufficiently addressed. They express a strong willingness to adapt their practice toward more sustainable models, provided scientific validation exists. Integrating environmental criteria into oncology decision-making and research frameworks will be important for the future sustainability of cancer care.

## 1 Introduction

Climate change represents one of the greatest threats to global health, with health-care systems contributing substantially to greenhouse gas emissions. In high-income countries, health care accounts for approximately 5–10% of national emissions [[1], [2], [3]], and cancer care is among the most resource-intensive domains due to frequent imaging, systemic therapies, surgical interventions, radiotherapy, and patient travel. Global estimates indicate that in 2023 there were 18.5 million new cancer cases and 10.4 million cancer-related deaths, contributing to 271 million disability-adjusted life-years (DALYs) worldwide, with a disproportionate burden in low- and middle-income countries [4]. The projected increase in cancer incidence and deaths up to 2050 underscores the urgent need for effective and sustainable cancer-control strategies, including attention to the environmental footprint of oncology care [2,3].

Cancer care poses a paradox: while striving to improve survival and quality of life, oncology relies heavily on carbon-intensive interventions. The environmental burden of oncology care paradoxically exacerbates some of the very conditions it seeks to treat. For instance, air pollution is responsible for up to 10% of lung cancer cases in France, illustrating the cyclical interplay between environmental degradation and cancer incidence [5,6]. Recent studies have quantified this burden, showing that systemic therapies such as immune checkpoint inhibitors and intravenous monoclonal antibodies (mAb), radiotherapy, and surgical interventions are among the highest contributors to healthcare-related greenhouse gas emissions [[7], [8], [9], [10]].

Even diagnostic procedures and routine hospital services, although individually less intensive, contribute substantially to the carbon footprint of oncology care [11,12]. In Australia, diagnostic imaging alone was shown to represent a substantial carbon burden, with PET-CT and MRI being among the most energy-intensive procedures [11]. Surgical and radiotherapeutic interventions also exhibit significant environmental footprints [7,13,14]. Industry-sponsored clinical trials, which are frequent in oncology, further contribute to emissions through complex logistics, travel, and data management infrastructures [15,16].

Addressing this “treat and harm” balance requires to understand how clinicians and other oncology professionals perceive their environmental responsibilities, and how such awareness could translate into actionable strategies. Existing literature has primarily focused on system-level or policy interventions to mitigate healthcare emissions, while data on individual perceptions and attitudes in oncology remain scarce [15,17]. Surveys among US physicians highlight growing recognition of ethical responsibilities in reducing the environmental impact of clinical practice. It shows that most physicians acknowledge climate change as a health issue and are willing to incorporate environmentally informed decisions, although practical integration remains limited [[18], [19], [20]]. However, these data are drawn from physicians across all specialties, and no study to date has specifically explored these questions within the oncology field.

Our study aims to fill this gap by assessing the perceptions of oncology healthcare professionals in France and Belgium regarding the environmental impact of cancer care, their awareness of the associated ethical tensions, and their willingness to adopt sustainable practices in clinical decision-making.

## 2 Methods

### 2.1 Study design and population

We performed a cross-sectional survey among oncology healthcare professionals in France and Belgium. Eligible participants included physicians, nurses, pharmacists, and allied health professionals involved in the diagnosis, treatment, coordination, or supportive management of patients with cancer. The survey was designed and conducted following the CHERRIES (Checklist for Reporting Results of Internet E-Surveys) guidelines to ensure methodological rigor and transparency. The survey was disseminated over 4 months (January 15 to April 15, 2025) via national oncology societies, social media platforms (mainly LinkedIn and X), and poster campaigns within hospital oncology departments.

### 2.2 Survey instrument

The 23-item questionnaire was developed by a multidisciplinary team including oncologists, nurses, and environmental health specialists. The development of the questionnaire was informed by existing literature on healthcare-related carbon emissions and sustainable oncology practices, as well as previously published surveys exploring healthcare professionals’ perceptions of climate change. It comprised five sections: Demographics and professional background; Perceived environmental impact of oncology; Integration of environmental criteria into decision-making; Willingness to modify practice; Expectations regarding evidence and communication formats. The survey also included a final open-ended question allowing respondents to suggest additional strategies not captured in the predefined items (Supplementary). The questionnaire underwent pilot testing among 10 oncology professionals from different disciplines (physicians, nurses, and allied health professionals) to assess clarity, relevance, and comprehensiveness of the items. Participants were asked to provide feedback on question wording, interpretation, and overall structure. Based on their feedback, minor revisions were made to improve clarity, reduce ambiguity, and ensure the appropriateness of response options.

### 2.3 Data analysis

Descriptive statistics were used to summarize responses. Continuous variables were expressed as means (SD) or medians (IQR), and categorical variables as counts (percentages).

Further statistical analyses were conducted using parametric methods. Student’s *t*-tests were used to compare mean values between two independent groups. When comparisons involved more than two groups, one-way analysis of variance (ANOVA) was applied. In cases where the ANOVA indicated a significant effect, post hoc comparisons were performed to identify group differences. Linear regression analyses were used to examine associations between continuous variables and to estimate the magnitude and direction of these relationships. Model assumptions were checked prior to analysis. Statistical significance was set at *p* < 0.05.

### 2.4 Ethical considerations

The survey was anonymous and non-interventional, in accordance with French and Belgian research regulations. No identifiable data was collected.

## 3 Results

### 3.1 Respondent characteristics

A total of 330 healthcare professionals completed the survey. Their characteristics are listed in Table 1. Respondents were predominantly women (73%), with a median age of 39 years (range 18–67). Professions included: medical oncologists (32%), nurses (25%), advanced practice nurses (8%), and other professionals engaged in any aspect of cancer care delivery, including pharmacists, radiologists and imaging technologists, laboratory scientists (35%). Most participants worked in France (83%). Institutions included mainly comprehensive cancer centers (45%) and university hospitals (29%).

**Table 1:** Respondents characteristics.Min: minimum. Max: maximum.

Characteristic	All respondents (N = 330)
**Gender**, n (%) Female / Male	241 (73) / 88 (27)
**Age, median (min-max)**	39 years (18–67)
**Profession, n (%)** Medical oncologist Nurse Advanced practice nurse Radiation therapist Resident Surgeon Hematologist Other (> 10 professions)	107 (32) 81 (25) 25 (8) 15 (5) 14 (4) 8 (2) 7 (2) 73 (22)
**Country, n (%)** France Belgium	271 (83) 56 (17)
**Institution type** Comprehensive cancer centers University hospitals Other	147 (45) 95 (29) 84 (26)

### 3.2 Perceptions of oncology’s environmental impact

Two-thirds of the respondents perceived oncology as having a higher environmental impact than other medical specialties (33.1% “significantly higher,” 37.7% “slightly higher”). This result was not influenced by any demographic parameters (gender, age, occupation, professional experience, region of practice, type of center).

**Fig. 1:**
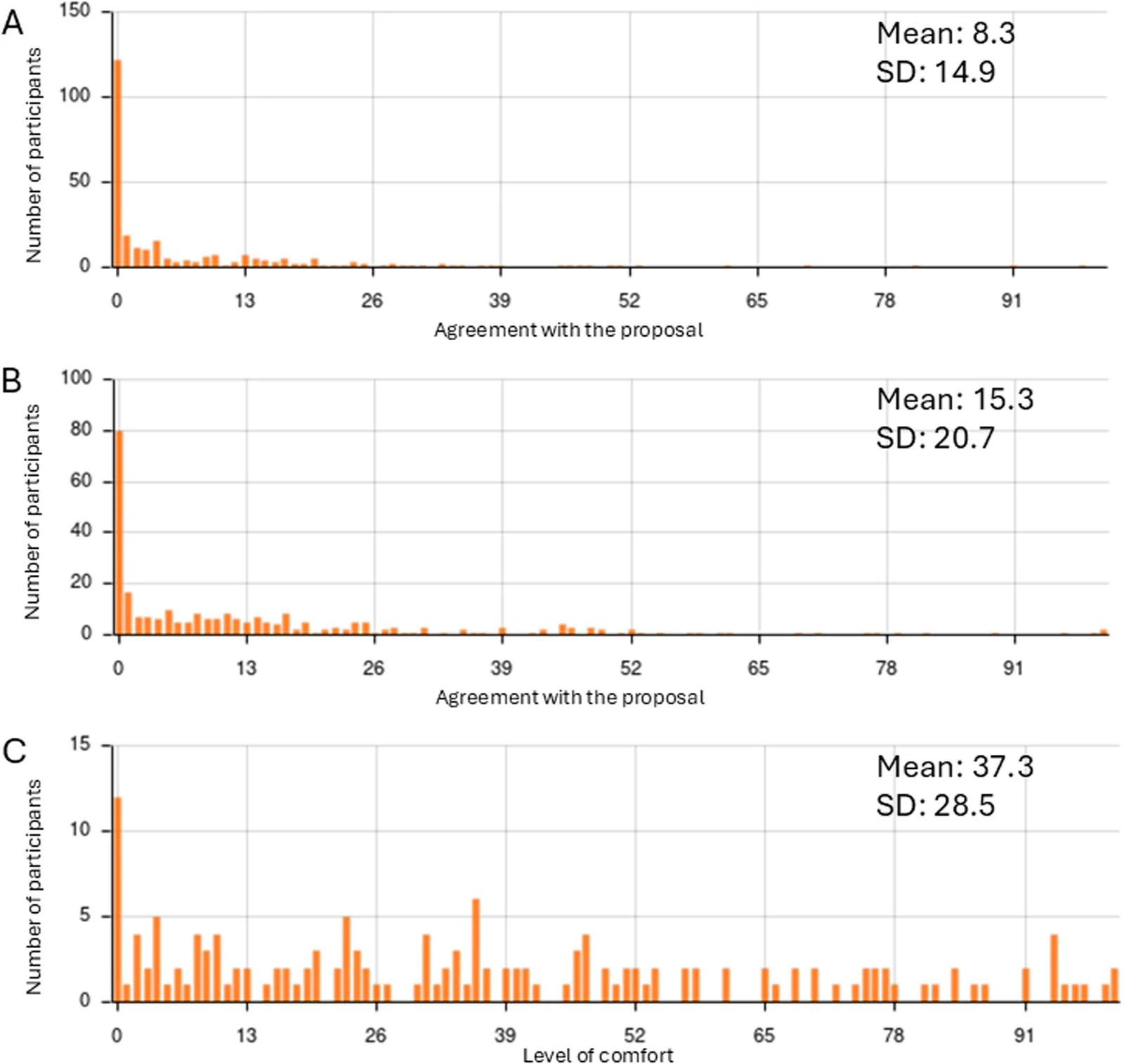
Histogram of the repartition of the votes regarding the place of the environmental impact in the medical decision regarding oncological care (A), the efforts done to decrease the environmental impact oncological care (B) and their degree of comfort facing this topic (C). All three questions were ranked from 0 to 100. A: At present, to what extent do you think the environmental impact of oncology care is considered in medical decision-making? (0: not at all; 100: completely) B: At present, do you consider the efforts made to reduce the environmental impact of oncology care to be sufficient? (0 = not at all; 100 = completely) C: How comfortable do you feel discussing or addressing the environmental impact of oncology care? (0: Not at all comfortable; 100: Very comfortable) SD: standard deviation.

The respondents estimated that the environmental considerations were rarely integrated into daily decision-making (mean 8.3/100, SD 14.9), and institutional initiatives were judged to be insufficient (mean 15.3/100, SD 20.7) (Fig. 1A and B).

Globally, respondents felt insufficiently informed about the environmental impact of oncology care (mean 2.7/6, SD 1.5). They also found the available resources for finding information on this topic too limited (mean 1.9/6, SD 1.2) and thought that national and international efforts on this topic were insufficient (mean 2.0/6, SD 0.94).

When asked about their level of comfort discussing the environmental impact of cancer care, respondents generally reported feeling uncomfortable, with substantial interpersonal variability (mean 37.3/100, SD 29.5) (Fig. 1C).

Factors influencing level of comfort discussing the environmental impact of cancer care were age (p < 0.001) and professional experience (p < 0.001); the youngest respondents and/or those with the least professional experience expressing the lowest level of comfort (Supplementary figure 1A). No significant differences were found regarding the type of center, region, gender, or profession, although residents seemed to express a lower level of comfort, which is consistent with the above results (Supplementary figure 1B).

### 3.3 Identified sources of environmental burden

Respondents identified the following items as major contributors to the environmental footprint of cancer care (ranked from most to least impactful): chemotherapy preparation and administration; use of biotherapies; radiotherapy sessions; diagnostic imaging; repeated patient transport; surgical procedures and hospital heating. In their own professional activities, participants mentioned travel to conferences, daily commuting, information technology (IT) equipment, and hospital consumption as key impact sources.

### 3.4 Attitudes toward sustainable practices

A large majority (97.7%) stated they would adopt lower-impact clinical strategies if equivalence in oncologic efficacy was demonstrated. When choosing between two equivalent therapeutic strategies in terms of efficacy and toxicity, participants ranked the following factors as those they would prioritize: patient quality of life (average rank [between 1 and 8] ± standard deviation: 1.86 ± 1.14), side effects (2.15 ± 1.18), cost (4.66 ± 1.57); lower environmental impact (4.79 ± 1.47); hospital routines (5.85 ± 1.63) or ease of use (5.76 ± 1.49). Respondents highlighted the need for robust scientific validation before changing practice and would be willing to choose a treatment strategy with less environmental impact if equivalence to the standard were demonstrated by large national or international cohorts (74.9%); randomized equivalence trials (67.7%); retrospective cohorts (39.5%); health insurance data analyses (32.3%).

All of the public policy actions suggested in the survey were considered to have the potential to significantly reduce the environmental impact of cancer care. These included implementing more systematic cancer screening programs (mean: 8.2/10, SD: 2.5); banning advertisements for fatty or sugary foods on television (mean: 8.8/10, SD: 2.6); promoting physical activity in public health policies (mean: 9.6/10, SD: 1.8); conducting anti-smoking campaigns (mean: 9.8, SD: 1.6); conducting campaigns against alcohol consumption (mean: 9.6/10, SD: 1.7); and promoting vaccination against influenza, HPV and hepatitis (mean: 9.4/10, SD: 2.0).

**Fig. 2:**
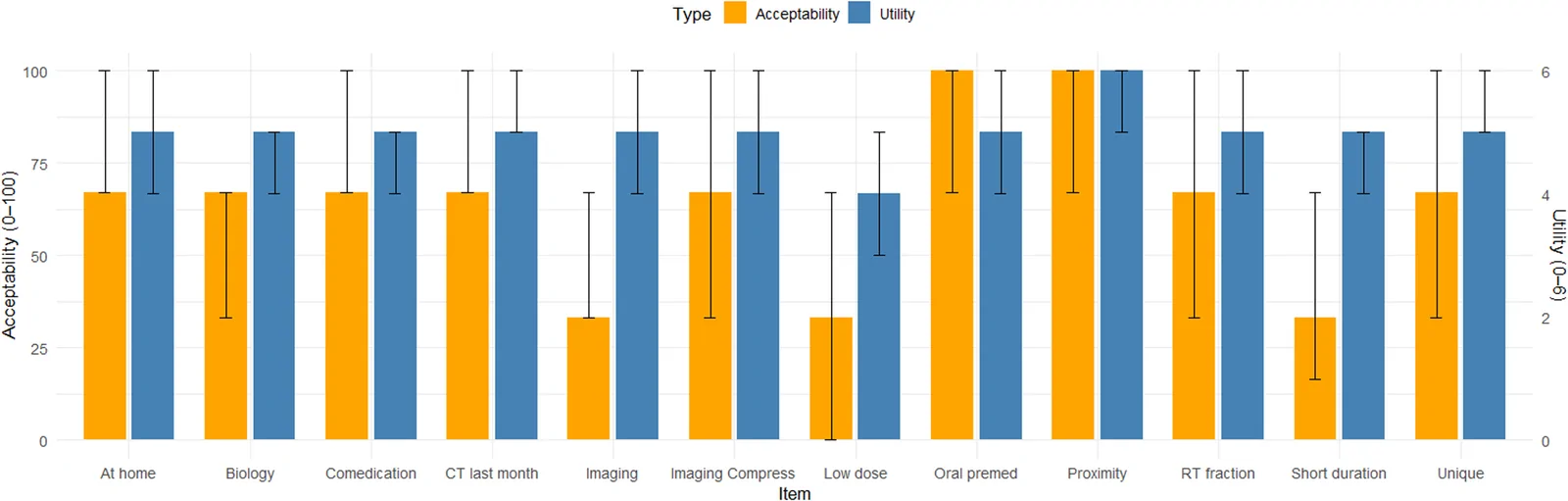
Histogram presenting the utility and the acceptability of 12 suggestions to decrease the environmental impact of oncological care, as ranked by the respondents. All results are presented by median and interquartile range. In orange: Acceptability. In blue: utility. At home: promote home-based chemotherapy administration (hospital-at-home programs); Biology: limit laboratory testing during treatment and follow-up; Comedication: rationalize patients’ co-medications; CT last month: limit chemotherapy administration during the last month of life by involving palliative care teams earlier; Imaging: limit imaging during follow-up; Imaging compress: discontinue or compress imaging after 10 years, keeping only written reports; Low dose: reduce the dose of certain treatments (e.g., immunotherapy); Oral premed: prefer oral rather than IV premedication for chemotherapy; Proximity: use local treatment centers closer to patients’ homes (chemotherapy or radiotherapy); RT fraction: reduce the number of radiotherapy fractions; Short duration: shorten the duration of certain treatments (e.g., immunotherapy); Unique: reduce the use of single-use medical devices.

A list of 12 suggestions to be implemented in the treatment strategies was addressed to the respondents to ascertain their vision about their utility and the acceptability (Fig. 2). Some were seen as highly useful and acceptable (oral instead of intravenous premedication, performing oncological systemic treatments in hospital near the patient’s place of living); others were seen as useful but potentially less acceptable (shortening of the systemic treatment duration, limiting the number/frequency of imaging or decreasing the dose of certain systemic treatment) (Fig. 2).

**Fig. 3:**
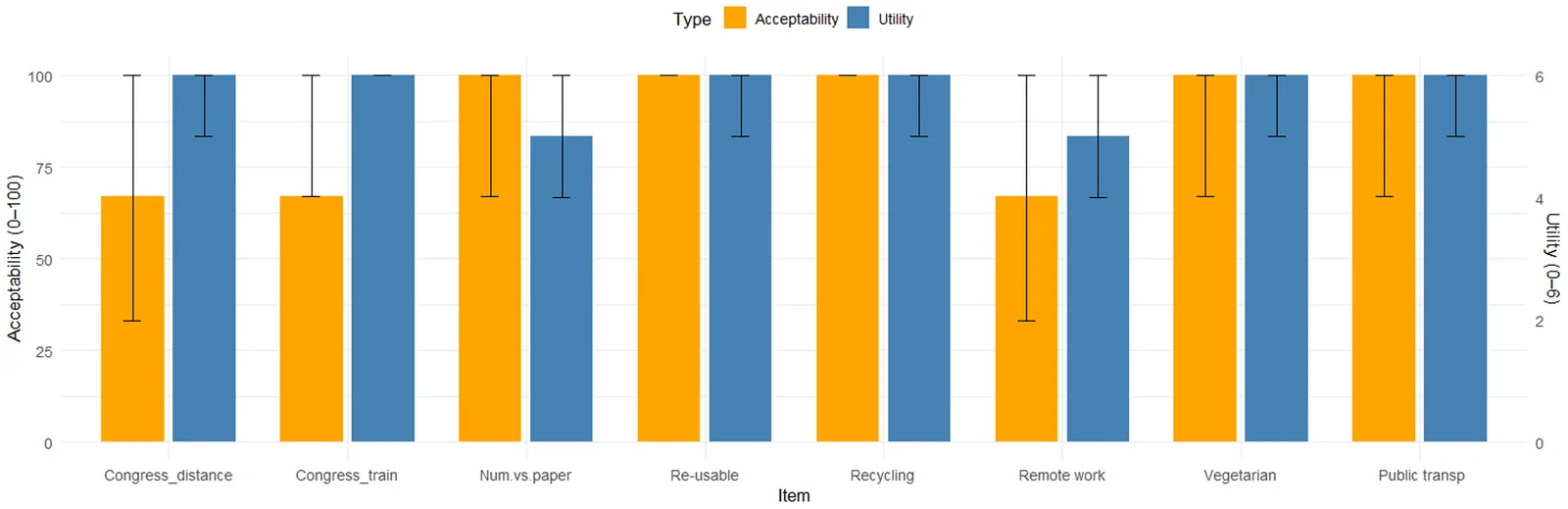
Histogram presenting the utility and the acceptability of 8 suggestions to implement in routine practice, as ranked by the respondents. All results are presented by median and interquartile range. In orange: Acceptability. In blue: utility. Congress_distance: attend conferences virtually rather than in person; Congress_train: take the train instead of flying to conferences; Num.vs.paper: prefer digital rather than printed materials; Re-usable: prefer reusable food containers (e.g., glass cups instead of cardboard); Recycling: implement waste sorting/recycling in your unit; Remote work: promote remote work when possible; Vegetarian: choose vegetarian meals more often at the hospital; Public transp: use bicycle or public transport instead of car.

A list of 8 suggestions to be implemented in daily practice was addressed to the respondents to reflect their vision about their utility and acceptability (Fig. 3). All the suggestions were seen as useful in terms of reduction of carbon footprint but some (preferring re-usable materials and implementing recycling in hospital) were seen as more readily acceptable than others (attending international conferences by train or participating remotely, or implementation of remote work) (Fig. 3).

### 3.5 Information and teaching

Preferred dissemination formats for sustainability information included professional websites (71.8%), conference presentations (71.8%), video webinars (43.5%), and newsletters (40%).

Dedicated learning and training by the medical faculties were also seen as a positive proposition (mean 5.1/6, SD 1.2)

### 3.6 Other suggestions made by respondents

The survey included a final open-ended question allowing respondents to suggest additional approaches. The suggestions made included concerns regarding unnecessary investigations in frail patients unlikely to receive active treatment (*“What is the ecological impact of extensive diagnostic workups in patients for whom no treatment will ultimately be given?”*), the need to *“return to clinical reasoning and semiology, which consume fewer examinations,”* and the environmental burden of digital infrastructures such as *“data storage, e-mails, and radiotherapy servers.”* Respondents also highlighted avoidable waste related to packaging and single-use materials (*“we sometimes open a pack of five compresses for only one compress”*), as well as the importance of territorial organization and proximity care (*“improve territorial coherence and care close to the patient’s home”*). Several comments stressed that ecological measures should remain compatible with healthcare organization and workload, emphasizing the need for *“financially viable systems”* and closer collaboration with hospital leadership. Finally, respondents called for stronger institutional and industrial accountability, including *“obtaining a specific carbon footprint for each therapeutic strategy and drug”* and *“imposing ecological requirements on pharmaceutical companies during drug development.”*

## 4 Discussion

This study provides novel insights into oncology professionals’ perceptions regarding the environmental impact of cancer care. Our findings reveal a strong awareness of oncology’s contribution to environmental degradation, accompanied by a willingness to change clinical practice when robust evidence supports alternatives. The gap between awareness and action highlights a perceived lack of standardized guidance and limited availability and awareness of validated data on the environmental consequences of specific oncologic interventions [1,17]. While national frameworks for sustainable oncology are emerging in countries such as the UK and the US, similar structured initiatives remain limited in French-speaking Europe, underscoring the importance of local engagement and policy development [21,22]. By linking professional attitudes to emerging data on the carbon footprint of oncologic interventions, we seek to provide exploratory insights into oncology professionals’ perceptions that may help inform future research, discussions, and actions for the development of more sustainable oncology practices, aligned with the principles of “green oncology” [23].

In our questionnaire (Supplementary Materials), we used the example of the care pathway of a patient with BRCA-mutated triple-negative breast cancer (including imaging, neoadjuvant chemotherapy with immunotherapy, surgery, radiotherapy, adjuvant PARP inhibition, and follow-up) to document respondents’ perceptions of the relative environmental impact of the various components of the care pathway. Climate change related to carbon emission is one dimension of the environmental burden of cancer care pathways for which data are available in the literature. Imaging alone contributes meaningfully, with a mammography generating approximately 0.5–0.8 kg of CO₂ equivalent (kg CO₂eq), a breast MRI around 17.5 kg CO₂eq, and a PET scan roughly 20 kg CO₂eq (based on Australian data, were electricity production is mainly (64% in 2024) based on fossil energy) [11,24]. Systemic therapy adds considerably to this burden, as intravenous immunotherapy accounts for approximately 325 kg CO₂eq per month, conventional chemotherapy <50 kg CO₂eq per month, and oral PARP inhibitors around 126 kg CO₂eq per month [8,11,25]. Procedural interventions further increase emissions, with surgery producing approximately 100 kg CO₂eq and a typical course of radiotherapy involving 25 fractions contributing up to 1200 kg CO₂eq [7,13]. Recent large-scale lifecycle assessment data further refine the environmental footprint of radiotherapy. In a multi-institutional analysis of external beam radiotherapy, Lichter et al. estimated that a standard 25-fraction course generates approximately 4,310 kg CO₂eq per treatment course, substantially higher than previously reported estimates. It is important to note that these data are derived from North American settings, where the energy mix differs significantly from that of France (primarily nuclear energy, which is carbon-free) and other European countries, which may partly explain discrepancies. Notably, the environmental burden was predominantly driven by facility energy consumption (≈74%) and patient transport (≈26%), while consumables contributed only marginally (<1%). Importantly, simulation models demonstrated that hypofractionated regimens could reduce greenhouse gas emissions by up to 42% in breast cancer and 77% in genitourinary cancers without compromising clinical intent, highlighting fractionation strategy as a key lever for decarbonisation in radiotherapy practice [26].

Some simple changes in the administration of drugs with the greatest environmental impact can already have a significant impact. For instance, Malmberg et al. demonstrated that treatment with pembrolizumab and nivolumab in a Dutch hospital generated approximately 445 tons of CO₂ equivalent in a single year, primarily driven by pharmaceutical production [8]. Their analysis further showed that implementing alternative dosing strategies (such as weight-based adjustments or extended dosing intervals) could reduce emissions by up to 26% for pembrolizumab and 11% for nivolumab without compromising clinical efficacy. It was also shown that the subcutaneous route for mAb injection did not represent a sustainable strategy, since reduction in single use material was counterbalanced by the need of higher dose of antibody. This highlights how precise life cycle assessment can refine our perception about the environmental impact of a treatment strategy [9,10].

Even seemingly minor components of care add measurable emissions; a recent study in acute surgical patients demonstrated substantial overuse of laboratory tests, with unnecessary bloodwork generating nearly 1 kg CO₂eq per patient and a standard blood panel emitting over 300 g CO₂eq. This highlights how reducing low-value care can meaningfully contribute to environmental sustainability [27]. Emerging strategies include telemedicine for follow-up visits, consolidation of clinical trial infrastructure to reduce travel-related emissions, and implementation of low-carbon trial design frameworks [15,16,20].

This synthesis provides a framework for a hierarchical understanding of environmental impact, highlighting radiotherapy and systemic immunotherapy as among the highest contributors, followed by surgery and imaging. Conventional chemotherapy displays a lower impact in terms of carbon emission. Similarly, supportive care and low-intensity diagnostic procedures present comparatively lower carbon footprints, while administrative processes contribute only marginally [2,3]. Survey respondents generally had a good knowledge of which parts of the care pathway generate the most CO2 emissions, but it is worth noting that some aspects were not well known, such as the greater impact of immunotherapy and radiation therapy compared to conventional chemotherapy. Regarding perceptions of feasibility, our survey results suggest that interventions such as dose reduction or treatment de-escalation are perceived as less feasible or less acceptable by oncology professionals (Fig. 2), despite their potential environmental benefit. This perception likely reflects a framing of these strategies as a reduction in quality of care rather than as a re-evaluation of treatment intensity based on evidence. In reality, when supported by robust clinical data, dose optimization or shortened treatment duration may offer multiple patient-centered benefits, including reduced hospital visits, decreased toxicity, improved quality of life, and potentially equivalent efficacy in selected indications. This highlights an important educational gap, where environmental considerations are not yet fully integrated into broader concepts of value-based care.

Although the survey was mainly based on a predefined list of mitigation strategies, it also featured a final open-ended question inviting participants to propose additional measures. The free-text responses broadened the range of potential mitigation approaches by revealing themes that were not addressed in the structured questionnaire. These suggestions included limiting low-value care (such as unnecessary diagnostic tests or treatments offering minimal clinical benefit), streamlining logistics and supply chains, tackling the environmental footprint of digital infrastructures, enhancing waste and resource management, and strengthening the accountability of the pharmaceutical industry.

However, this qualitative component was limited to a single open-ended question and did not allow for an in-depth exploration of these perspectives, which should be addressed in future research using dedicated qualitative methodologies. Several respondents also highlighted that sustainability efforts should prioritize system-level and organizational interventions rather than relying solely on individual behavioral changes. This approach may help reduce environmental impact without increasing clinicians’ cognitive or operational burden, supporting the implementation of sustainable practices in routine care. As an illustration, some of the terms used in our survey (e.g., “limit” or “rationalize”) may have been perceived as restrictive. However, these concepts align with existing principles of value-based care and the reduction of low-value interventions. In this context, environmentally sustainable oncology should not be viewed as a constraint but rather as an opportunity to improve the quality, efficiency, and appropriateness of care.

These responses, along with the low assessment of the feasibility of certain strategies (such as reducing the dose or duration of certain treatments) show the emerging ethical tensions the oncology community faces (as do other medical disciplines), between maximizing individual patient benefit and ensuring collective environmental responsibility. Most study participants did not feel comfortable with the issue of the environmental impact of oncology care, especially those who were young and/or had little professional experience. They overwhelmingly supported evidence-based ecological transitions rather than symbolic or unilateral restrictions. These findings are consistent with broader surveys showing that healthcare professionals recognize their ethical responsibilities regarding climate change but often lack practical tools to implement sustainable practices [[18], [19], [20]]. However, these findings should be interpreted with caution, as the survey likely attracted participants already sensitized to or interested in environmental issues, introducing a selection bias that may overestimate the overall level of awareness and engagement among oncology professionals. Interestingly, the group categorized as “other caregivers” appeared to report higher levels of comfort in discussing environmental issues. This group includes heterogeneous profiles such as pharmacists, radiology staff, and laboratory professionals, who may have greater exposure to system-level processes or environmental management practices. Their relative comfort may also reflect both differences in training and a lower degree of direct involvement in patient-facing therapeutic decision-making, as well as more limited exposure to the clinical trade-offs inherent to oncology practice (Supplementary figure 1B). Educational initiatives, institutional commitment, and inclusion of environmental indicators in clinical research and health policy remain critical next steps. Future health technology assessments (HTA), which traditionally evaluate clinical efficacy, safety, and cost-effectiveness, should also incorporate environmental impact metrics. Greenhouse gas emissions can be converted into future health loss (expressed in QALYs or DALYs) providing a meaningful way to balance the environmental cost against the health benefit of a care pathway. Recent estimates suggest that approximately 66.4 tCO₂e (95% CI: 21.7–148.1) are associated with one future year of life lost [6]. Integrating life-cycle assessment data into HTA would enable decision-makers to consider the carbon cost of oncologic interventions alongside their therapeutic value, promoting reimbursement and policy frameworks that align medical benefit with environmental sustainability [13,28]. Acknowledging environmental risk factors for cancer reinforces the need of sustainable oncology practices within the broader context of planetary health and cancer prevention [5,29]. Current oncology practices have largely been established based on clinical efficacy, safety, and cost-effectiveness, with little consideration given to their environmental impact. As a result, these practices have become accepted as the “standard of care” without integrating sustainability as a core component of quality. In this context, environmentally responsible strategies should not be viewed as alternatives to standard care, but rather as an extension of value-based care principles, aiming to optimize outcomes while minimizing unnecessary resource use and environmental harm.

## 5 Conclusion

Oncology professionals in France and Belgium acknowledge that the environmental impact of cancer care is substantial and insufficiently addressed. However, perceived feasibility remains a major barrier, particularly for strategies such as dose reduction or treatment de-escalation, which are often interpreted as reductions in quality of care rather than as evidence-based optimization strategies. Overall, our study highlights that while awareness and willingness to act are high, there remains a gap in the accurate perception of relative environmental impacts across oncology practices and in the acceptance of certain evidence-based de-escalation strategies as legitimate components of value-based care. Finally, bridging the gap between awareness and action requires robust data, national frameworks, and educational programs to support environmentally sustainable cancer care.

## Funding

No specific funding was received beyond AERIO’s (Association pour l'Enseignement et la Recherche des Internes en Oncologie [the French Association of Residents in Oncology]) support for survey hosting.

## CRediT authorship contribution statement

**Paul Matte:** Writing – original draft, Methodology, Data curation, Conceptualization. **Pierre-Florent Petit:** Writing – review & editing, Methodology. **Anthony Turpin:** Writing – review & editing. **Caroline Sarre-Pradon:** Writing – review & editing. **Adrien Rousseau:** Writing – review & editing. **Marc Hilmi:** Writing – review & editing. **Sarah Dumont:** Writing – review & editing. **Pauline Parent:** Writing – review & editing. **Anne Rullier:** Writing – review & editing. **Eva Ester Molina-Beltran:** Writing – review & editing. **Max Piffoux:** Writing – review & editing, Validation. **Matthieu Delaye:** Writing – review & editing, Visualization, Methodology, Formal analysis, Conceptualization.

## Declaration of competing interest

The authors declare that they have no competing interests related to this work or its publication.

## Footnotes

Supplementary material associated with this article can be found, in the online version, at doi:10.1016/j.joclim.2026.100741.
