# Supplementary material for: Perceptions of the environmental impact of oncology care: A cross-sectional survey of French and Belgian healthcare professionals
Source: J Clim Chang Health. 2026 Sep 10;31:100741. doi: 10.1016/j.joclim.2026.100741 (PMC13583773; doi:10.1016/j.joclim.2026.100741)
Supplement: Supplementary file 1 [file mmc2.pdf]

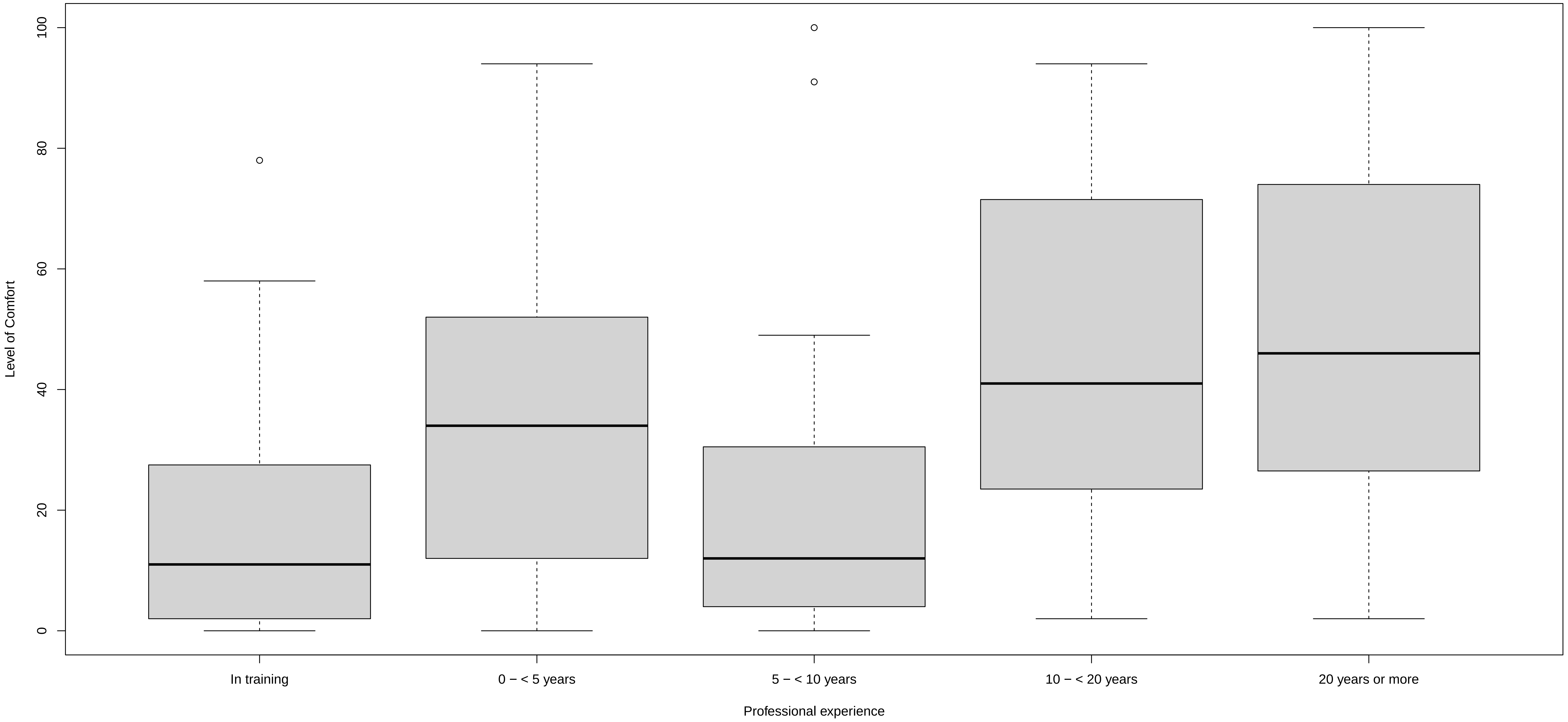
Level of Comfort
0
20
40
60
80
100
In training
0 − < 5 years
5 − < 10 years
10 − < 20 years
20 years or more
Professional experience
