# Supplementary material for: Perceptions of the environmental impact of oncology care: A cross-sectional survey of French and Belgian healthcare professionals
Source: J Clim Chang Health. 2026 Sep 10;31:100741. doi: 10.1016/j.joclim.2026.100741 (PMC13583773; doi:10.1016/j.joclim.2026.100741)
Supplement: Supplementary file 2 [file mmc3.pdf]

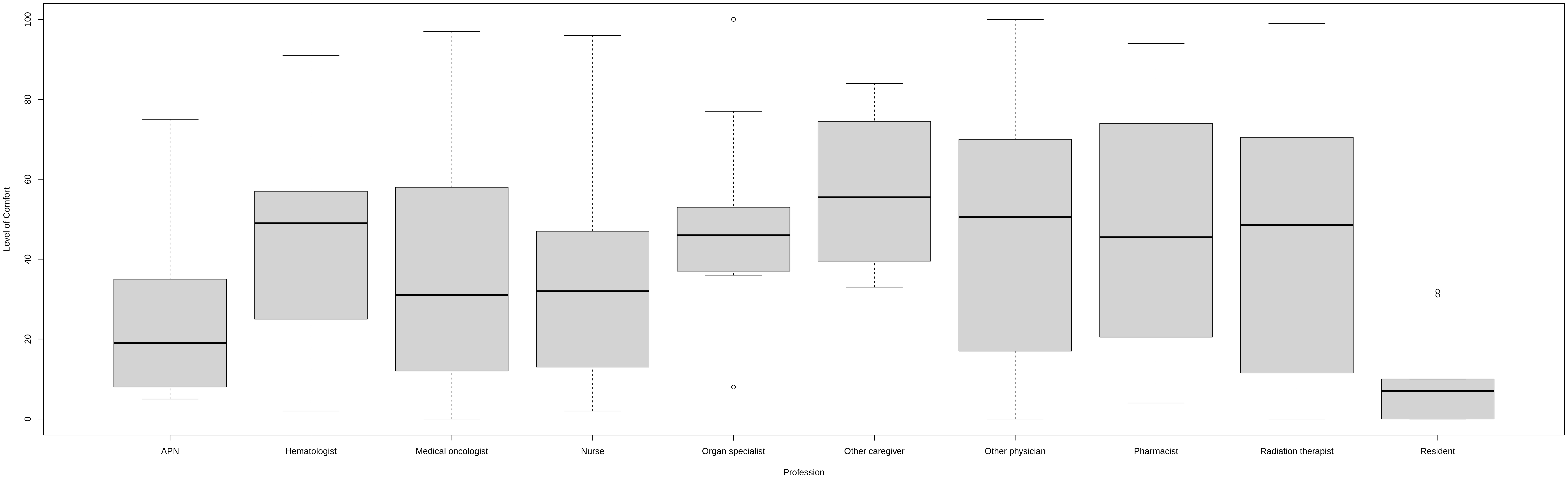

Level of Comfort
0
20
40
60
80
100
APN
Hematologist
Medical oncologist
Nurse
Organ specialist
Other caregiver
Other physician
Pharmacist
Radiation therapist
Resident
Profession
