# Supplementary material for: Perceptions of the environmental impact of oncology care: A cross-sectional survey of French and Belgian healthcare professionals
Source: J Clim Chang Health. 2026 Sep 10;31:100741. doi: 10.1016/j.joclim.2026.100741 (PMC13583773; doi:10.1016/j.joclim.2026.100741)
Supplement: Supplementary file 3 [file mmc1.docx]

**Supplementary material:**

- **Supplementary figure 1: Boxplots showing the level of comfort regarding the environmental impact of oncology care according to professional experience (1A) and profession (1B).**
  APN: advanced practice nurse.
- **Translated questionnaire.**
