# Supplementary material for: Perceptions of the environmental impact of oncology care: A cross-sectional survey of French and Belgian healthcare professionals
Source: J Clim Chang Health. 2026 Sep 10;31:100741. doi: 10.1016/j.joclim.2026.100741 (PMC13583773; doi:10.1016/j.joclim.2026.100741)
Supplement: Supplementary file 4 [file mmc4.docx]

**Survey on the perception of environmental impact in oncology**

Dear participant,

Thank you for taking a few minutes to complete this survey.

Its aim is to assess the level of awareness among healthcare professionals involved in cancer care in Europe, regarding the environmental impact of oncological practices, and to identify areas that may require further research and/or education.

This is an exploratory survey, there are no right or wrong answers.
Its goal is to foster reflection on the environmental footprint of cancer management. Completing it should take approximately 15 minutes.

Your contribution is valuable, and we sincerely thank you for your time.

1) **Participant profile**

**What is your gender?**

- Male
- Female
- Other
- Prefer not to say

**What is your age?**

**What is your specialty?**

- Medical oncologist
- Radiation oncologist
- Organ-specific oncologist
- Hematologist
- Surgical oncologist
- Resident in one of these specialties
- Nurse
- Advanced practice nurse
- Other (please specify):

**In which region do you currently practice?**

- Auvergne–Rhône–Alpes
- Bourgogne–Franche–Comté
- Brittany
- Centre–Val de Loire
- Corsica
- Grand Est
- Hauts-de-France
- Île-de-France
- Normandy
- Nouvelle-Aquitaine
- Occitanie
- Pays de la Loire
- Provence–Alpes–Côte d’Azur
- French overseas territories
- Other French-speaking region
- French-speaking Belgium
- French-speaking Switzerland

**How long have you been practicing (since obtaining your medical degree)?**

- In training
- 0–<5 years
- 5–<10 years
- 10–<20 years
- ≥20 years

**What is your main practice setting?**

- University hospital
- General hospital
- Comprehensive cancer center
- Private institution
- Other (please specify):

**How would you describe the environment of your main practice site?**

- Densely urban
- Moderately urban
- Rural

**How would you describe the local medical density of your main practice area?**

- Low medical density
- Average medical density
- High medical density

2) **General perception of the environmental impact of oncology practice**

**To what extent do you consider that your current oncology practice has a negative impact on the environment?**
(0 = no impact; 100 = very high impact)

**Compared with other medical specialties, would you say that oncology has a negative environmental impact that is:**

- Much higher
- Slightly higher
- About the same
- Slightly lower
- Much lower

**At present, to what extent do you think the environmental impact of oncology care is considered in medical decision-making?**
(0 = not at all; 100 = completely)

**At present, do you consider the efforts made to reduce the environmental impact of oncology care to be sufficient?**
(0 = not at all; 100 = completely)

3) **Perception of the environmental impact of different phases of cancer care**

**Patient Case Study**

Imagine a 45-year-old woman diagnosed with a BRCA-mutated triple-negative breast cancer.
Her treatment plan includes several stages:

1. Staging workup including mammography, ultrasound, breast MRI, and PET-CT
2. Neoadjuvant chemotherapy: 6 cycles of intravenous chemo-immunotherapy
3. Breast surgery: partial mastectomy and sentinel lymph node biopsy
4. Radiotherapy: 25 sessions on the operated breast and regional lymph nodes
5. Adjuvant therapy: PARP inhibitor
6. Follow-up: regular clinical visits, blood tests, and imaging

Within this care pathway, please **rank the following activities** from **most to least polluting**, according to your perception:

- Biopsy (operating room, pathology materials)
- Administration of intravenous cytotoxic chemotherapy
- Use of targeted or immunotherapy (e.g., IV anti-PD1 in neoadjuvant setting, oral PARP inhibitor in adjuvant setting)
- Imaging procedures (mammography, ultrasound, MRI, PET-CT)
- Radiotherapy sessions
- Surgical procedures (partial mastectomy, sentinel node biopsy)
- Supportive care medications (in hospital and at home)
- Hospital heating and electricity consumption
- Use of medical informatics systems
- Patient transportation between home and treatment site
- Purchase of disposable medical devices (needles, syringes, ECG, etc.)
- Laboratory tests
- Patient’s daily life (food, energy, transportation, etc.)

**Physician Case Study**

Imagine a typical workday for an oncologist:

They drive 40 minutes to the hospital by car.
On arrival, they put on a clean lab coat each morning.
They conduct outpatient consultations using electronic medical records.
At lunch, they eat at the hospital cafeteria (meat or fish available daily).
They drink three cups of coffee in disposable cups during the morning.
In the afternoon, they attend a multidisciplinary tumor board, then complete patient files and work on research projects before driving home.
Twice a year, they fly to international conferences (ASCO in the U.S., ESMO in Spain).
The hospital building, constructed in the 1960s, has poor insulation, gas heating, and partial air conditioning in summer.

In this oncologist’s daily routine, please **rank the following activities** from **most to least polluting**, according to your perception:

- Hospital heating and electricity consumption
- Laundry management (cleaning hospital garments)
- Purchase, maintenance, and use of medical IT equipment
- Commuting to the hospital
- Food and coffee consumption
- Air travel for conferences

4) **Perception of the utility and acceptability of changes in cancer care**

For each of the following potential measures, please indicate:

- Their **potential impact** on reducing the environmental footprint of cancer care (--- = no effect; +++ = major reduction), and
- Their **acceptability** (-- = totally unacceptable; ++ = totally acceptable),
  assuming no randomized clinical trial data are yet available.

| **Proposed measure** | **Environmental impact** | **Acceptability** |
| --- | --- | --- |
| Prefer oral rather than IV premedication for chemotherapy |  |  |
| Reduce the number of radiotherapy fractions |  |  |
| Use local treatment centers closer to patients’ homes (chemotherapy or radiotherapy) |  |  |
| Reduce the dose of certain treatments (e.g., immunotherapy) |  |  |
| Shorten the duration of certain treatments (e.g., immunotherapy) |  |  |
| Limit imaging tests during follow-up |  |  |
| Limit laboratory testing during treatment and follow-up |  |  |
| Reduce the use of single-use medical devices |  |  |
| Rationalize patients’ co-medications |  |  |
| Limit chemotherapy administration during the last month of life by involving palliative care teams earlier |  |  |
| Discontinue or compress imaging after 10 years, keeping only written reports |  |  |
| Promote home-based chemotherapy administration (hospital-at-home programs) |  |  |

5) **Public health measures**

For each of the following, please rate their potential impact on reducing environmental footprint:
(0 = no effect; 10 = very high impact)

- Implement more systematic cancer screening programs
- Ban advertisements for fatty or sugary foods on television
- Promote physical activity in public health policies
- Conduct anti-smoking campaigns
- Conduct campaigns against alcohol consumption
- Promote vaccination (influenza, HPV, hepatitis, etc.)

**Would you agree to implement a lower environmental impact strategy if equivalent efficacy data were available?**

- Yes
- No

**What type of evidence would you require before adopting such a strategy?**

- Randomized equivalence trial
- Large national or international cohort data (e.g., ESME)
- Health insurance database analyses (e.g., SNDS)
- High-quality retrospective cohort
- Other (please specify):

**In case of oncologic-outcome equivalence demonstrated between two therapeutic strategies, rank the following arguments from most to least relevant in guiding your choice:**

- Lower rate of adverse events
- Improved quality of life
- Lower financial cost
- Lower environmental impact
- Institutional experience and familiarity with the treatment
- Ease of administration
- Patient comfort (oral treatment, fewer hospital visits)
- Patient’s personal choice

6) **Individual perceptions and practices**

Please rate the **potential environmental benefit** (--- to +++) and **acceptability** (-- to ++) of the following personal measures:

| **Measure** | **Environmental impact** | **Acceptability** |
| --- | --- | --- |
| Use bicycle or public transport instead of car |  |  |
| Take the train instead of flying to conferences |  |  |
| Attend conferences virtually rather than in person |  |  |
| Prefer digital rather than printed materials |  |  |
| Implement waste sorting/recycling in your unit |  |  |
| Choose vegetarian meals more often at the hospital |  |  |
| Prefer reusable food containers (e.g., glass cups instead of cardboard) |  |  |
| Promote remote work when possible |  |  |

7) **Education and level of awareness**

**How comfortable do you feel discussing or addressing the environmental impact of oncology care?**

- Not at all comfortable
- Very comfortable

**To what extent do you agree with the following statements?**
(--- = strongly disagree; +++ = strongly agree)

- I am sufficiently aware of the environmental impact of my oncology practice.
- Available sources of information on the environmental impact of oncology are adequate.
- International scientific societies (ASCO, ESMO, etc.) are making sufficient efforts to address ecological issues at their events.
- Medical schools should include dedicated teaching on the environmental impact of healthcare.

**Which formats of information on the environmental impact of oncology practice would you find most useful?**

- French-language website
- English-language website
- Regular newsletter
- Presentation at congresses
- Video webinar
- Other (please specify):

**In your opinion, what other sources of environmental footprint reduction have not been addressed in this survey?**

Please feel free to share any comments or leave your contact information if you wish to receive more information on this topic.
